# Functional study on new *FOXL2* mutations found in Chinese patients with blepharophimosis, ptosis, epicanthus inversus syndrome

**DOI:** 10.1186/s12881-018-0631-8

**Published:** 2018-07-20

**Authors:** Lu Zhou, Jiaqi Wang, Tailing Wang

**Affiliations:** The 3rd Department, Plastic Surgery Hospital of the Chinese Academy of Medical Sciences, Peking Union Medical College, Badachu Road, Shijingshan District, No. 33, Beijing, 100041 China

**Keywords:** BPES, FOXL2, Gene mutation

## Abstract

**Background:**

Blepharophimosis, ptosis, epicanthus inversus syndrome (BPES) is a rare inheritable disease that mainly affects eyelid development associated with (type I) or without (type II) ovarian dysfunction, resulting in premature ovarian failure (POF). Mutations in the gene forkhead box L2 (*FOXL2*) have been shown to be responsible for BPES. The aim of this study was to determine and functionally validate the *FOXL2* mutation in a Chinese BPES family.

**Methods:**

Twelve individuals including five BPES patients from a Chinese family were enrolled. Genomic DNA was extracted from peripheral blood of enrolled subjects. The coding region of the *FOXL2* gene was amplified and mutations were determined by sequencing analyses. Functional analysis was carried out to study changes in expression and transcriptional activity of the mutant FOXL2 protein.

**Results:**

A novel mutation in the *FOXL2* gene (c.931C > T) was detected in all five BPES patients, which converts a histidine residue into a tyrosine (p.H311Y) in the FOXL2 protein. Functional analysis revealed that this point mutation reduces FOXL2 protein expression, concomitant with decreased transcriptional activity on the steroidogenic acute regulatory (StAR) gene promotor.

**Conclusions:**

Our results expand the mutational spectrum of the *FOXL2* gene and provide additional insights to the research on the molecular pathogenesis of *FOXL2* in BPES*.*

## Background

Blepharophimosis, ptosis, epicanthus inversus syndrome (BPES, OMIM # 110100) is a rare genetic disorder with an estimated incidence of 1 in 50,000 births [1]. It can occur sporadically or associate with autosomal dominant mutations. The characteristic clinical presentations of this disease include a complex eyelid/ocular malformation characterized by blepharophimosis, ptosis, epicanthus inversus and telecanthus. The horizontal shortening of the palpebral aperture can lead to amblyopia in one or both eyes [2]. Depending on the occurrence of premature ovarian failure (POF) or not, female BPES patients are classified into two different groups, with type I patients having POF while type II referring to those with normal ovarian function [3]. However, both types of BPES are widely recognized to result predominantly from mutations in the forkhead transcriptional factor-2 (*FOXL2*) gene that is involved in palpebral and ovarian development [4].

The *FOXL2* gene in human is approximately 2.7-kb long located on chromosome 3q22.3, which encodes a protein with 376 residues. The FOXL2 protein contains a characteristic 100 amino-acid DNA-binding forkhead domain, which categorizes the FOXL2 protein into the superfamily of Forkhead transcription factors [5]. An alanine-rich domain, also known as a polyalanine (poly-Ala) tract highly conserved across various mammals, lies downstream of the forkhead domain and is responsible for negatively regulating its transcriptional activity [5]. FOXL2 localizes in the nucleus and transcriptionally modulates genetic programs required for early eyelid development and ovary differentiation and maintenance, at the same time, represses components essential for somatic testis determination [6]. FOXL2 acts as a transcriptional repressor for multiple genes, including the human steroidogenic acute regulatory (StAR) gene. StAR mediates the transports of cholesterol across mitochondrial membrane, and controls the rate-limiting step in steroidogenesis [7]. Mutations in *FOXL2* may contribute to de-repression of the StAR promoter and cause increased differentiation of granulosa cells, leading to the onset of POF [8].

A collection of more than 100 genetic alternations affecting the *FOXL2* locus have been identified in patients with BPES, including frameshifts, insertions, nonsense as well as missense mutations [9, 10], with intragenic mutations accounting for the majority (71%) [11]. Although there are some claims about the correlations between different mutations in FOXL2 and BPES types, direct genotype-phenotype association remains to be further demonstrated because of the lack of de novo genetic study using animal model and the clinical heterogeneity among patients with BPES [12]. Furthermore, the mechanisms underlying individual mutations causing the development of BPES are largely unknown and could be complicated. It is likely that some mutant FOXL2 might collaborate with other cellular alternations to promote disease progression, as exemplified by the coexistence of the FOXL2 deletion and BMP15 in some BPES patients [13]. Therefore, identification of novel *FOXL2* mutations and further characterization of their contributions to the pathogenesis of BPES will provide not only biomarkers for early detection of BPES but also potential implications for therapeutic intervention.

Here, we report a novel FOXL2 mutation identified in a Chinese family with BPES. Functional studies of this missense mutation (c.931C > T, p.H311Y) revealed a reduction in both FOXL2 protein expression and its transcriptional repression activity on the promoter of StAR gene, underscoring the significance of this mutation in the pathogenesis of BPES.

## Methods

### Patients

**Fig. 1:**
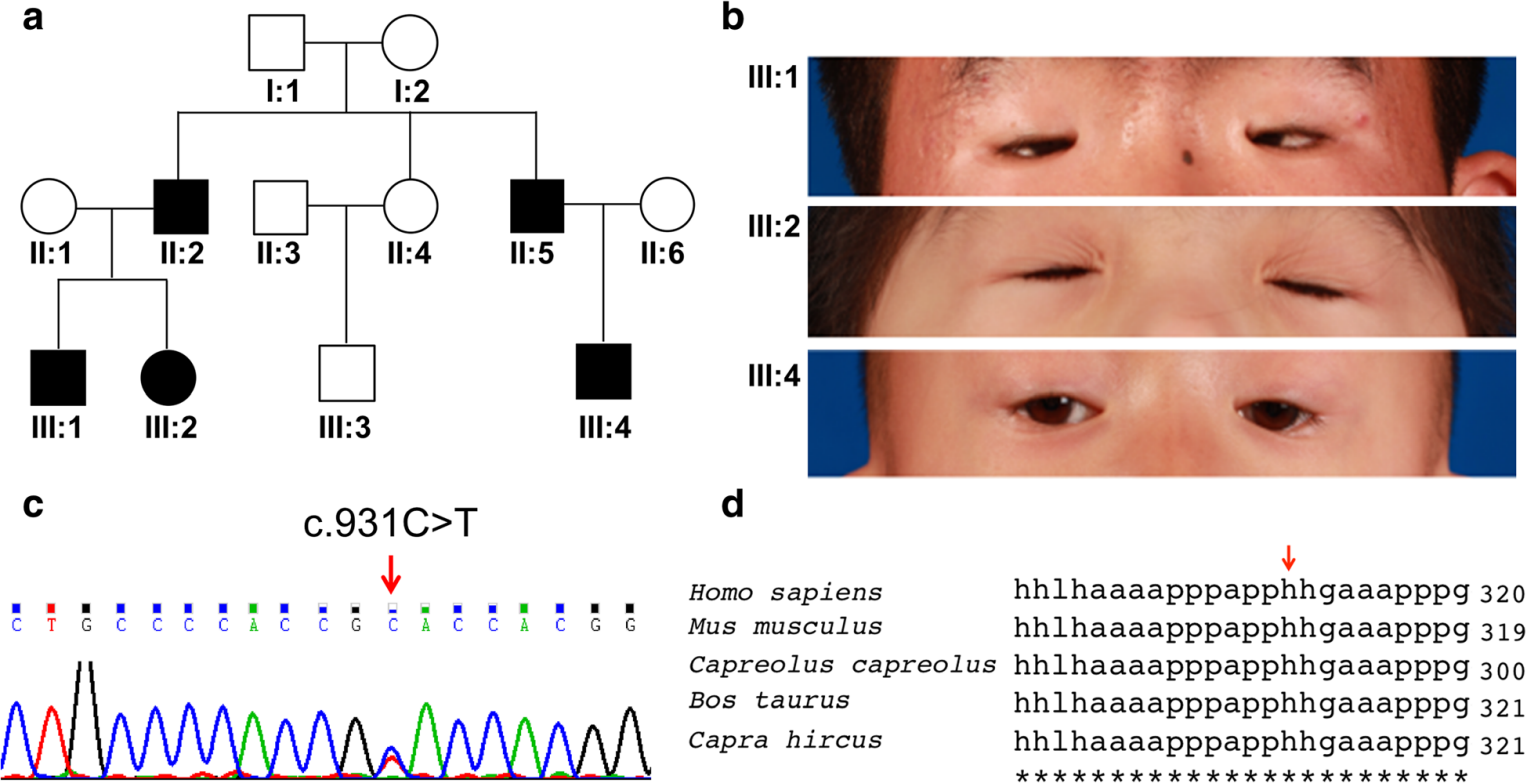
Detection of *FOXL2* mutation in BPES patients. **a**. The Pedigree of a Chinese family with BPES. **b**. Representative images of BPES patients. **c**. Sequencing result indicating the c.931C > T mutation in the *FOXL2* gene. **d**. Alignment of FOXL2 proteins from different species showing the conserved site of H311

A Chinese family with BPES was ascertained at the Plastic Surgery Hospital of Chinese Academy of Medical Sciences (Fig. 1a). A total of 12 individuals, including five with BPES were enrolled in this study. Clinical examinations of the patients were performed by ophthalmologists, following the criteria listed below to diagnose BPES [14]: blepharophimosis, ptosis, epicanthus inversus, and telecanthus. POF was determined if the patients underwent ovarian failure under the age of 40 and they presented features including amenorrhoea, hypoestrogenism and elevated serum gonadotrophin concentrations. This study was approved by the Ethics Committee of our institute, and informed consent was obtained from all participants or their guardians for research.

### DNA extraction and sequencing

Blood samples were collected from peripheral vein, followed by leukocytes enrichment and genomic DNA isolation using phenol and chloroform. The full-length FOXL2 open reading frame (ORF) was amplified by touch-down PCR with High-Fidelity Taq Polymerase (Invitrogen). After gel analysis to confirm the success of PCR, the amplicons were subjected to Sanger sequencing (Applied Biosystems), followed by nucleotide blasting to determine any mutation. Primers are available upon request.

### Tissue culture and DNA vectors

HEK293T cells and HeLa cells were obtained from ATCC and maintained in DMEM (Sigma-Aldrich) with 10% fetal bovine serum (FBS), 2 mM L-glutamine, 100 U/ml penicillin and 100 mg/ml streptomycin. The ovarian granulosa cell tumor KGN cells were cultured in DMEM-F12 medium with 10% FBS, 100 U/ml penicillin and 100 mg/ml streptomycin. All cells were cultured in humidified incubator at 37 °C with 5% CO_2_. Transfection was performed using Lipofectamine 2000 (Invitrogen) according to the manufacturer’s instruction. WT and mutant *FOXL2* (NM_023067) expression constructs are based on a pcDNA3.1 plasmid backbone. The FOXL2-H311Y expression vector was obtained using junction-PCR according to method as previously described [15].

### Cellular fractionation and immunoblotting

Cytosolic/nuclear fraction from cells transfected with different expression vectors was performed with Cell Fractionation Kit (Abcam), according to the manufacturer’s instruction. For whole cell lysate, cells were lysed in a buffer containing 1% IGEPAL, 150 mM NaCl, 20 mM HEPES (pH 7.9), 10 mM NaF, 0.1 mM EDTA, 1 mM sodium orthovanadate and 1× protease inhibitor cocktail. Protein concentration was quantified using BCA protein concentration assay kit (Pierce). Lysates were electrophoresed on SDS-polyacrylamide (SDS-PAGE) gels and proteins were then transferred to nitrocellulose membrane (Millipore). Membranes were incubated with primary antibodies (FOXL2, actin, Lamin B1) in 5% bovine serum albumin containing 0.05% Tween-20 at 4 °C overnight, followed by incubation with HRP-conjugated secondary antibody at room temperature for 1 h, and visualization by an ECL or ECL Prime (GE Healthcare) [16].

### Luciferase assay

For luciferase assays, HeLa cells and KGN cells were seeded in 24-well plates. Transient co-transfection was carried out with indicated combinations of luciferase reporter vectors (pGL2-basic, pGL2-StAR:− 1300 bp-luciferase [17] and RSV-Renilla) and the control pcDNA 3.1 expression vector. A Renilla reporter driven by an RSV promoter (Promega) was used as a control for transfection efficiency. After incubation for 24 h at 37 °C, the transfected cells were washed 3 times with phosphate buffered saline (PBS) and lysed by passive lysis buffer, followed by Dual-luciferase assay according to manufacturer’s instructions (Promega). Transfection was conducted in triplicate and experiments were performed at least three times. Relative luciferase unit is the ratio of Firefly over Renilla luciferase read. Statistical significance was determined by non-parametric Mann-Whitney test.

### Quantitative real-time PCR

RNA was prepared using Trizol reagent (Agilent Technologies) and Direct-zol RNA MiniPrep Kit (Zymo Research) according to the manufacturer’s instructions and cDNA was generated using high capacity cDNA reverse transcription kit (Applied Biosystems) with random primers [18]. RT-qPCR was performed with SYBR Green (Qiagen) using primers as described previously [19].

### Statistics

Graph Pad Prism 5.0 was used to perform non-parametric Mann-Whitney test to compare all interval variables. Error bars express +/− standard error of the mean.

## Results

### Clinical findings

All five patients from the affected family in this study demonstrated the typical features of BPES, including small palpebral fissures, ptosis of the eyelids, telecanthus and epicanthus inversus (Fig. 1a, b). Only one female patient was identified, with no sign of POF, probably due to the young age.

### Genetic findings

Sequencing analysis of the *FOXL2* locus from the affected individuals revealed a heterozygous missense mutation c.931C > T (p.H311Y) (Fig. 1c), which has never been reported in familial BPES and is absent in the 100 ethnically matched control chromosomes. The C > T mutation causes a single amino acid substitution at the residue 311, converting a histidine residue into a tyrosine (p.H311Y). Interestingly, the histidine 311 residue of FOXL2 protein is highly conserved across species (Fig. 1d) while the Grantham distance score (83) between histidine and tyrosine is high [20], suggesting this amino acid substitution might have a functional impact on FOXL2 protein and subsequently the pathogenesis of BPES.

### Expression level of mutant FOXL2

**Fig. 2:**
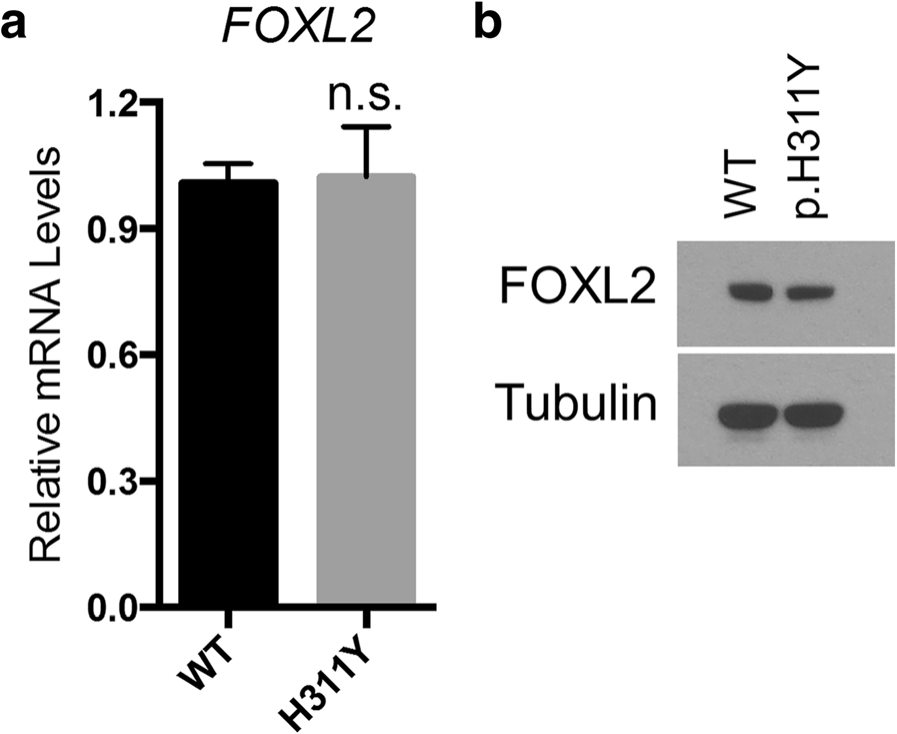
Expression levels of WT and p.H311Y mutant *FOXL2*. **a**. mRNA expression level of *FOXL2*, as measured by qPCR, was comparable between WT and p.H311Y mutant. **b**. Protein level of *FOXL2*, as determined by Western Blot, showed lower p.H311Y expression when compared to WT. n.s.: not significant

To test whether the c.931C > T (p.H311Y) mutation influences the functions of FOXL2 protein, we generated expression vectors for both wild-type (WT) and mutant (p.H311Y) FOXL2. Upon transfection into HEK293T cells, the expression of FOXL2 can be detected at both mRNA and protein levels (Fig. 2). Whereas the transcription of FOXL2 was comparable between cells transfected with WT and mutant constructs (Fig. 2a), the WT FOXL2 protein was more abundant than the mutant one (Fig. 2b), suggesting that this point mutation affects the stability of the FOXL2 protein.

### Subcellular localization of mutant FOXL2

**Fig. 3:**
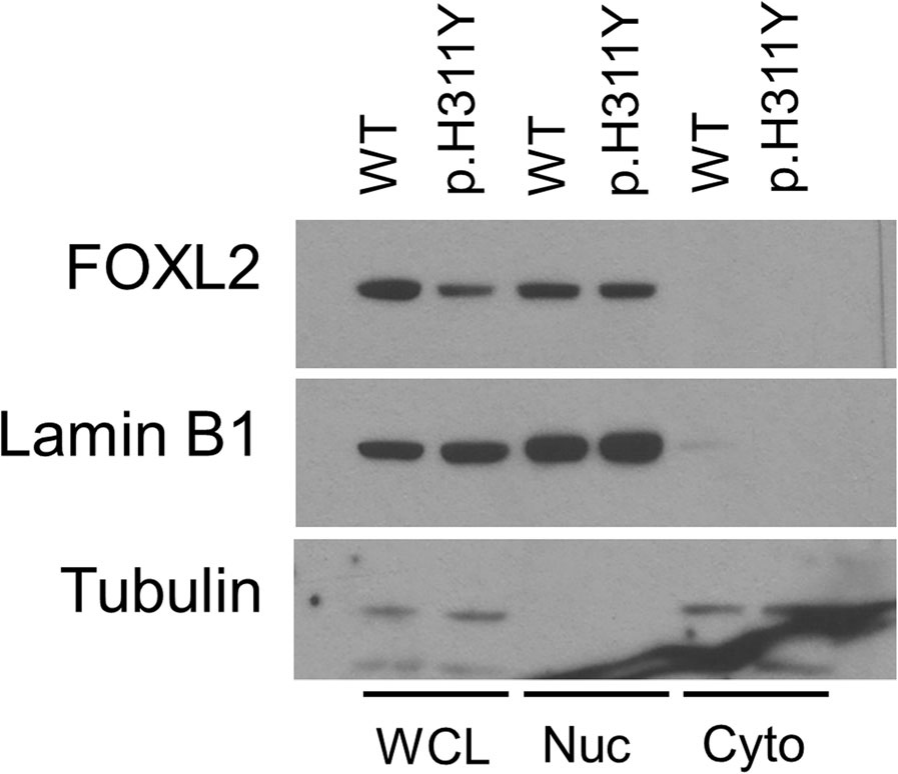
Subcellular distribution of WT and p.H311Y mutant *FOXL2*. Nuclear (Nuc) and cytosolic (Cyto) extracts, as well as whole cell lysate (WCL) of cells transfected with WT or p.H311Y FOXL2 were subjected to Western blotting. Both WT and mutant FOXL2 showed nuclear localization. Lamin B1 and tubulin were used as markers of nuclear and cytoplasmic fractions, respectively

Given that FOXL2 functions as a transcription factor predominantly localizing in the nucleus, we continued to examine whether p.H311Y mutation influences the subcellular localization of the FOXL2 protein. Both WT and mutant FOXL2 localized exclusively in the nucleus (Fig. 3), suggesting that the p.H311Y mutation does not alter the cellular distribution pattern of FOXL2.

### Transcriptional activity on StAR promoter

**Fig. 4:**
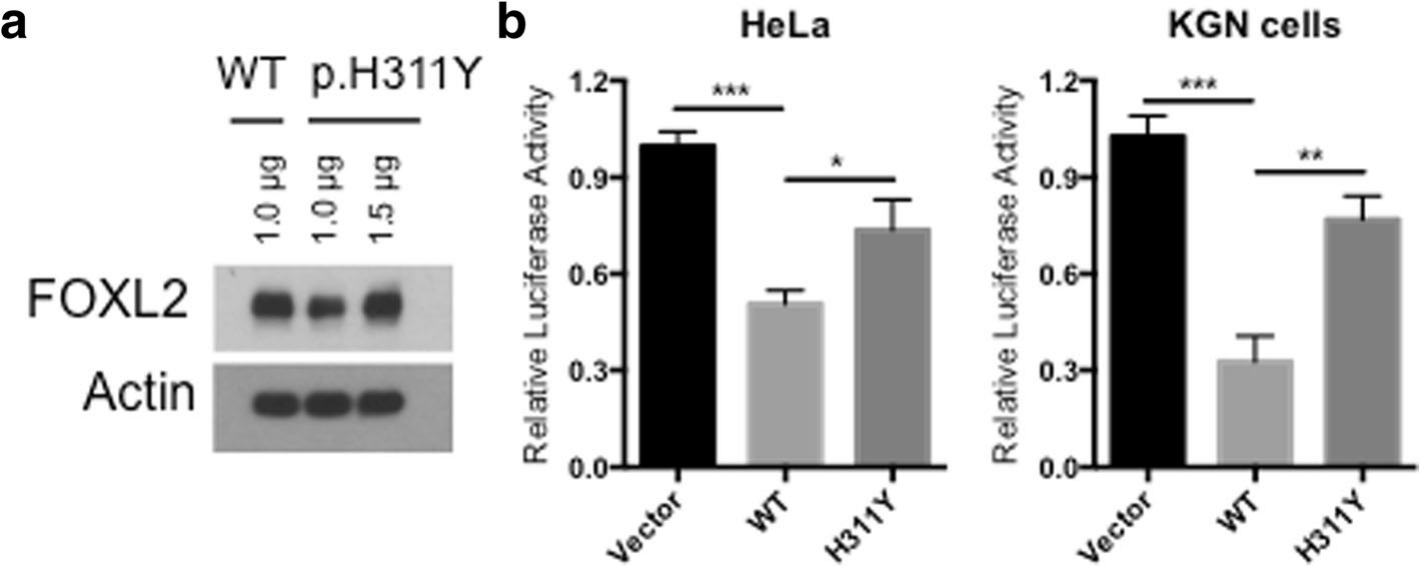
Transcriptional repression activity of WT and p.H311Y mutant *FOXL2*. **a**. Expression level of WT and p.H311Y mutant FOXL2. **b**. Transcriptional repression activity of WT and p.H311Y mutant FOXL2 as measured by luciferase assay in both HeLa and KGN cells. Luciferase vector driven by the StAR promoter was cotransfected with empty vector, WT or p.H311Y mutant FOXL2 plasmids, followed by luciferase activity measurement. *p < 0.05, **p < 0.01

To confirm whether the missense mutation alters the transcriptional activity of FOXL2, we performed luciferase-based reporter assays to assess their transactivation capacity on promoter of StAR, a well-characterized target of FOXL2 [8, 21]. Given that the mutant FOXL2 is less stable than its WT counterpart, we first titrated the amount of DNA for transfection so as to obtain similar protein level (Fig. 4a). As expected, WT FOXL2 repressed the StAR promoter activity as reflected by decreased luciferase intensity (Fig. 4b). In contrast, cells transfected with the same amount of mutant FOXL2 construct (p.H311Y) did not show significant inhibition of the StAR promoter activity (Fig. 4b), suggesting the loss of FOXL2 function upon p.H311Y mutation.

## Discussion

In this study, we report a novel missense mutation in the *FOXL2* gene from a Chinese family with BPES. Additionally, we functionally characterized the effects of this mutation on FOXL2 activity, experimentally validating the relevance of this mutation to the pathogenesis of BPES.

Genetic alternation in *FOXL2* locus has been long appreciated as an important causal factor for the pathogenesis of BPES. The highly conserved FOXL2 protein is a transcription regulator containing a DNA-binding forkhead domain and a poly-Ala tract, both of which host the vast majority of BPES-associated mutations identified to date [9]. The novel mutation c.931C > T causes a single amino acid substitution at the residue 311, converting a histidine residue into a tyrosine (p.H311Y). Although located outside the forkhead domain and poly-Ala tract, the histidine 311 residue of FOXL2 protein is highly conserved across species (Fig. 1c). Given that a histidine residue is nucleophilic and usually serves a role in stabilizing the folded structures of proteins while a tyrosine residue is hydrophobic and can make a protein exceedingly unstable when ionized, the replacement of histidine by tyrosine is likely to alter the functional property of FOXL2 protein. Indeed, further tests showed that although the c.931C > T (p.H311Y) mutation does not alter mRNA levels of FOXL2; it greatly decreases its protein expressions, probably by reducing the stability of FOXL2 protein.

Both WT and mutant FOXL2 localized exclusively in the nucleus, suggesting that the p.H311Y mutation does not affect the protein’s nuclear localization signal or disturb interactions with nuclear transporters. A previous study has reported that mutations in the forkhead domain of FOXL2 are more likely to cause cytoplasmic mislocalization and aggregation of the protein [22]. The c.931C > T (p.H311Y) mutation occurs outside the forkhead and the poly-Ala domains, and does not seem to alter the subcellular localization and aggregation pattern of FOXL2. This is confirmed by another report by Beysen et al. that a missense mutation located outside the forkhead domain (p.S217F) had no effect on intracellular protein distribution [23]. Interestingly, luciferase assay of the StAR gene showed loss of FOXL2 repressor function upon p.H311Y mutation as compared to the WT FOXL2. This could be due to a decrease of total available FOXL2 in the nucleus. It is also possible that the mutation may decrease intranuclear mobility, as well as binding affinity of FOXL2 to the StAR gene. Further studies are required to corroborate these assumptions.

Given the critical roles of FOXL2 mutations in the development and progression of BPES, enormous efforts have been made in the correlations between genotypes and phenotypes. Initial studies have been focused more on the FOXL2 structural alternations and ended with a preliminary genotype-phenotype correlation, that is, mutations resulting in truncated FOXL2 proteins without the poly-Ala tract are likely associated with BPES type I, whereas poly-Ala expansions might preferentially give rise to BPES type II [12]. However, clear correlation seems difficult for the mutations contributing to mutant proteins but with an intact forkhead domain and poly-Ala tract. Therefore, the functional properties of the affected FOXL2 proteins, including their transactivation capacity, subcellular localization, aggregation as well as protein 3D structure, are taken into account when classifying pathogenic FOXL2 mutations [23–25]. For example, using luciferase reporter systems, Dipietromaria et al. were able to demonstrate that loss-of-function FOXL2 mutants are likely BPES type I mutations [24]. Based on this theory, it is speculated that mutations outside the forkhead domain without affecting the FOXL2 transactivation are possibly associated with BEPS type II. Indeed, this seems true when analyzing all the known non-forkhead missense mutations. Including this novel one reported by our study, there are six different non-forkhead missense FOXL2 mutations: p.K193C, p.Y215C, p.S217C, p.S217F, p.Y258N and p.H311Y. Among these mutations, p.K193C was identified in a type II family [9]; p.Y215C, p.S217C and p.S217F have been experimentally validated using the dual 4xFLRE-luc and SIRT1-luc reporter systems and classified as BPES type II [24]. Although the individual carrying the p.Y258N mutation displayed POF, she was not a BPES patient [26]. As for our novel p.H311Y mutation, we are not able to tell its phenotype association at this point. On one hand, the single female patient in this affected family is still too young to pathologically classify the BPES subtype; on the other hand, the experimental approach we employed in this study was completely different than those used by Dipietromaria et al. (StAR-luciferase construct rather than the dual 4xFLRE-luc/SIRT1-luc reporters) [24], making it impossible to compare the transactivation capacity of p.H311Y. Therefore, whether or not our novel p.H311Y belongs to the BEPS type II still deserves further experimental investigation and clinical follow-up.

Furthermore, the severe phenotype associated with p.H311Y mutation is somehow consistent with other published missense mutations located outside the forkhead domain. For example, a heterozygous *FOXL2* missense mutation c.C650G (p.S217C) identified in an Iranian family with BPES gives rise to a striking phenotype with bilateral amblyopia [27]. Interestingly, the same mutation (p.S217C) has also been reported in an Indian family with mild eyelid phenotype [22], implying that additional genetic and/or epigenetic variations between these two p.S217C families might underlie the discrepancy in disease presentation. Despite that *FOXL2* located on 3q23 is the only gene known to cause BPES, considering the previous reports that the whole segment of 3q21–24 contributes to BPES [28], and that FOXL2 deletion coexists with BMP15 variants in a BPES patient [13], it is reasonable to speculate that other genetic and/or epigenetic factors synergize mutant FOXL2, especially those with intact transactivation capacity, to cause and/or exacerbate BPES. Further investigation on this aspect will be of interest, as it might reveal some novel components that are therapeutically targetable.

## Conclusions

In summary, we not only identified a novel mutation, c.931C > T (p.H311Y), in the *FOXL2* gene in a Chinese family with BPES, but also confirmed that this missense mutation causes a reduction in both the expression and the activity of FOXL2 protein. The novel mutation reported here further expands the mutation spectrum of the *FOXL2* gene and contributes to the understanding of the molecular pathogenesis of BPES.

## Abbreviations

BPES: Blepharophimosis-ptosis-epicanthus inversus syndrome
FOXL2: Forkhead box L2
ORF: Open reading frame
PCR: Polymerase chain reaction
POF: Premature ovarian failure
Poly-Ala: Poly-alanine
StAR: Steroidogenic acute regulatory
WT: Wildtype
